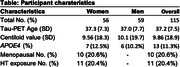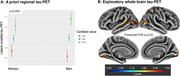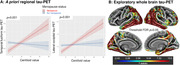# Sex, menopause, and hormone therapy moderate the PET tau and Aβ association in cognitively unimpaired adults with Down Syndrome: Findings from the Alzheimer Biomarkers Consortium — Down Syndrome

**DOI:** 10.1002/alz.086293

**Published:** 2025-01-09

**Authors:** Gillian T Coughlan, Ziwen Yuan, Elizabeth J. Andrews, Rory Boyle, Mabel Seto, Stephanie A. Schultz, Keith A Johnson, Reisa A Sperling, Michael J Properzi, Beau Ances, Elizabeth Head, Rachel F. Buckley

**Affiliations:** ^1^ Massachusetts General Hospital, Harvard Medical School, Boston, MA USA; ^2^ University of California, Irvine, Irvine, CA USA; ^3^ Harvard Medical School, Massachusetts General Hospital, Boston, MA USA; ^4^ Harvard Medical School, Boston, MA USA; ^5^ Gordon Center for Medical Imaging, Department of Radiology, Division of Molecular Imaging and Nuclear Medicine, Massachusetts General Hospital, Harvard Medical School, Boston, MA USA; ^6^ Center for Alzheimer’s Research and Treatment, Brigham and Women’s Hospital/Harvard Medical School, Boston, MA USA; ^7^ Department of Neurology, Massachusetts General Hospital, Harvard Medical School, Boston, MA USA; ^8^ Washington University in St. Louis School of Medicine, St. Louis, MO USA; ^9^ Brigham and Women's Hospital and Department of Neurology, Massachusetts General Hospital, Harvard Medical School, Boston, MA USA

## Abstract

**Background:**

Virtually all adults with Down Syndrome(DS) show Alzheimer’s disease(AD)‐related pathologic change by the age of 40 years. While sex differences in Aβ‐dependent tauopathy are apparent during early sporadic AD, sex differences in the DS population remain under‐investigated. Moreover, menopause onset occurs earlier in the DS population (45 years), and it remains unknown whether menopause status and hormone therapy(HT) exposure influences Aβ‐dependent tauopathy in women with DS. In a cognitively unimpaired DS population, we investigated cross‐sectional associations between Aβ and regional tau as a function of sex, menopause‐status, and HT‐exposure.

**Method:**

115 cognitively unimpaired individuals from the Alzheimer Biomarkers Consortium—Down Syndrome (Mean Age 37.9; 56 women [48%]; 13 *APOE*ε4 carriers [11%];Table 1) underwent Pittsburgh Compound‐B/Florbetapir(Aβ‐PET) and Flortaucipir(tau‐PET). Global Aβ was transformed to centiloid scale. 10 (20.6%) women self‐reported as being menopausal. 11 (20.4%) women reported HT exposure. Four *a priori* tau regions previously demonstrating sex differences in sporadic AD were selected (entorhinal cortex, inferior temporal gyrus, fusiform gyrus, lateral occipital cortex). Linear regressions (covarying age) examined the sex*Aβ interaction for each tau‐PET outcome. Similar models were examined in the subset of women, investigating menopause‐status[not menopausal/menopausal]*Aβ and HT*Aβ interactions. Exploratory whole‐brain vertex‐wise tau‐PET analyses were conducted with sex*Aβ and menopause*Aβ (modelled‐separately) and a FDR threshold *p*=0.05.

**Result:**

The sex*Aβ interaction showed a trend level association with tau‐PET, suggesting men exhibit elevated posterior‐temporal and lateral‐occipital tau with higher Aβ, relative to women (Figure 1). The menopause status*Aβ analyses indicated that menopausal women with higher Aβ exhibit significantly elevated temporal, lateral occipital and parietal tau (Figure 2). Sensitivity analyses covarying an age*Aβ interaction suggested that the menopause‐tau association was not driven solely by advancing age. Finally, higher temporal fusiform(p=0.020) and lateral occipital(p=0.004) tau‐PET signal was observed in women with HT‐exposure at higher levels of Aβ, relative to women without exposure.

**Conclusion:**

Sex differences in the Aβ‐tau association were marginal and require additional investigation. Menopause‐status and HT‐exposure influenced the association between Aβ and regional tau. While our results lack statistical power and should be replicated in larger DS populations, the findings suggest that sex‐specific biomarker profiles in DS may help determine sex‐specific pathways and hormonal mechanisms underlying increased risk of dementia.